# Hydrocyanic Acid in Mania

**Published:** 1864-11

**Authors:** 


### HYDROCYANIC ACID IN MANIA.

A celebrated doctor of mental disease, Dr. MacLeod, having been led to investigations by the use which has been made of the cherry-laurel water, and of other matters containing cyanogen, in mania, and thinking that their ineffectiveness resulted from their mode of preparation, experimented with prussic acid even in that frequent form of mental aberation, and his attempts were crowned with success. When writers say that there is no therapeutic treatment for mental diseases, and put forward manual labor as the best thing that could be done, it is necessary to answer these opinions by proofs of the contrary. M. Brierre of Boismont, whose word cannot be suspected of partiality in this matter, has already made protestations against these imprudent assertions, and it would be desirable that all those who think the same would say so. It is for this reason that we give Dr. MacLeod's report.

Out of forty cases which he has experimented upon, there were thirteen of acute mania, and four of chronic mania; two of puerperal mania, and one of intermittent mania; four with epileptiform fits, two of which were accompanied with menstrual derangements; rwo with hemiplegia; five with general paralysis; one with chronic hydrocephalus. Altogether thirty-four cases of mania, and six with acute or chronic melancholia with over-excitement. Eight of these different cases reported at full length allow us to appreciate their character and their severity.

The preparation that was chosen was constantly Scheele's acid diluted, at a dose of from two to five drops, either in a watery solution internally, or added to thirty drops of water in subcutaneous injections with Wood's syringe.

Beyond this dose accidents might occur, and it is prudent to stop at five drops. If it does not take effect rapidly the dose can be repeated, and if the over-excitement, after having disappeared, re-appears, a second dose will surely quiet it. The interval of these repeated doses must vary, according to the nature of the case, from five to fifteen minutes, as long as it has not produced its effect. It can be of one or two hours when its action wants to be kept up, and then it can be left to the judgment of an intelligent nurse.

In every case this remedy has acted when the natural course of the disease, its etiological effects as well as the diet, the moral treatment, and other causes acting simultaneously with it, have also been considered. Acting on the mind, and consisting principally in the gradual cessation of excitement, with or without sleep, it has never failed, although varying in intensity and in durability according to the case. Therefore it was slower, lighter in mania and acute and chronic melancholia with organic lesions, than in the opposite case, where it was immediate and continuous. It was also instantaneous in the violent fits of epileptiform and menstrual mania, and in the acute paroxysms of melancholia.

The effect was immediate when, for example, a patient was roaring, jumping, swearing, he became quiet, sat down, and sometimes fell into a deep sleep, from one to five minutes, after the administration of the remedy; gradual when the paroxysms were diminished, distant, anticipated, and that the patient became more reasonable, sociable and docile. These mental manifestations having reached a degree evident to everybody, and acknowledged by the patients themselves, are independent of any physical phenomenon. Twice only the pulse became slower, weaker, and slightly irregular, which perhaps was owing to the difficulty of observing it in such cases. The dose having been given too strong in two other cases, it produced coma with adynamia, foam at the mouth, difficulty in breathing and quick pulse, as before an epileptic fit. Slight headache, with nausea, and a special constriction of the throat, with involuntary incapacity of motion, were felt in other cases a few minutes after having taken the medicine.

In the forty cases in question the effect of the medicine was light, ten times temporary—that is to say, that the amelioration was only for a time without any action on the cause of the disease. The patients ceased to be violent, uneasy, noisy, excited, destructive, became more tractable, and a great deal better inclined for a moral and dietetic treatment. This result has been observed in a case of perpetual mania where the dose of the remedy had been insufficient, and in two cases of acute mania and melancholia where its use was not continued. In three acute manias and one perpetual mania the intensity of the disease soon made it fatal, and in two recent manias the effect, although real, was completed by other means, and a cure was obtained.

Nineteen times the action was more pronounced and permanent, though the disease remained stationary or progressed. Such were the five cases of general paralysis, five chronic manias, and three melancholias, whose acut-paroxysms were dissipated by these means. The same way in a case of insanity with great excitement, and four epilepsies, two of which had very prolonged fits under the influence of menstruation, one hysterical mania. and one perpetual mania, in which tranquility and sleep were obtained where other means had failed, and two other manias, with hemiplegia and hydrocephalus.

This medicine has, on the contrary, acted a most useful part in the rapid cure of eight cases, six of which were acute manias, and two melancholias. It has therefore great advantages by the rapidity, certainty and simplicity of its calming effects, the facility of its use, and the absence of any consecutive accidents. It use is indicated in all cases of mental diseases with over-excitement as an antagonist of this pathological phenomenon, without, however, preventing the simultaneous use of other means of cure. It is therefore superior to baths, opium and blood-letting, which it is designed to supercede.—*Dub. Med. Press*, May 4, 1864, from *Journ. de Med. de Bruxelles.—Med. News.*

